# Asymmetric Interaction Between Two Mycorrhizal Fungal Guilds and Consequences for the Establishment of Their Host Plants

**DOI:** 10.3389/fpls.2022.873204

**Published:** 2022-06-09

**Authors:** Natalia Fernández, Tereza Knoblochová, Petr Kohout, Martina Janoušková, Tomáš Cajthaml, Jan Frouz, Jana Rydlová

**Affiliations:** ^1^Laboratorio de Microbiología Aplicada y Biotecnología, Centro Regional Universitario Bariloche, Universidad Nacional del Comahue - IPATEC, Bariloche, Argentina; ^2^Consejo Nacional de Investigaciones Científicas y Técnicas (CONICET), Buenos Aires, Argentina; ^3^Department of Mycorrhizal Symbioses, Institute of Botany, Czech Academy of Sciences, Průhonice, Czechia; ^4^Institute of Microbiology, Czech Academy of Sciences, Prague, Czechia; ^5^Department of Experimental Plant Biology, Faculty of Science, Charles University, Prague, Czechia; ^6^Faculty of Science, Institute for Environmental Studies, Charles University, Prague, Czechia

**Keywords:** arbuscular mycorrhizae, ectomycorrhizae, mycorrhizal networks, primary succession, *Hieracium caespitosum*, *Betula pendula*

## Abstract

Arbuscular mycorrhiza (AM) and ectomycorrhiza (EcM) are the most abundant and widespread types of mycorrhizal symbiosis, but there is little and sometimes conflicting information regarding the interaction between AM fungi (AMF) and EcM fungi (EcMF) in soils. Their competition for resources can be particularly relevant in successional ecosystems, which usually present a transition from AM-forming herbaceous vegetation to EcM-forming woody species. The aims of this study were to describe the interaction between mycorrhizal fungal communities associated with AM and EcM hosts naturally coexisting during primary succession on spoil banks and to evaluate how this interaction affects growth and mycorrhizal colonization of seedlings of both species. We conducted a greenhouse microcosm experiment with *Betula pendula* and *Hieracium caespitosum* as EcM and AM hosts, respectively. They were cultivated in three-compartment rhizoboxes. Two lateral compartments contained different combinations of both host plants as sources of fungal mycelia colonizing the middle compartment, where fungal biomass, diversity, and community composition as well as the growth of each host plant species’ seedlings were analyzed. The study’s main finding was an asymmetric outcome of the interaction between the two plant species: while *H. caespitosum* and associated AMF reduced the abundance of EcMF in soil, modified the composition of EcMF communities, and also tended to decrease growth and mycorrhizal colonization of *B. pendula* seedlings, the EcM host did not have such effects on AM plants and associated AMF. In the context of primary succession, these findings suggest that ruderal AM hosts could hinder the development of EcM tree seedlings, thus slowing the transition from AM-dominated to EcM-dominated vegetation in early successional stages.

## Introduction

Soil microorganisms affect litter decomposition and nutrient release. They also can directly and indirectly influence the composition and productivity of plant communities, having significant effects on seedling establishment and vigor as well as on overall plant fitness (van der Heijden et al., 2008; Lau and Lennon, 2012; Peay, 2016). In terms of biomass and the ecosystem processes that they perform, some of the most important microbial groups in soil are mycorrhiza-forming fungi. Mycorrhizae usually exist as mutualistic symbioses between soil fungi and the roots of most terrestrial plants wherein fungal-foraged soil nutrients are exchanged for plant-derived photosynthates (Smith and Read, 2008; Brundrett and Tedersoo, 2018). Mycorrhizae benefit host plants by enhancing water and nutrient uptake and by increasing host resistance to pathogens and other biotic and abiotic stresses (Smith and Read, 2008; Brundrett and Tedersoo, 2018). The hyphae that extend from the roots into the soil enable the formation of mycorrhizal networks (MNs), which are composed of continuous fungal mycelia linking two or more plants of the same or different species. Mycorrhizal networks contribute to soil stabilization and aggregation (Smith and Read, 2008), and they positively influence seedling establishment and development (Nara, 2006a,b; Gorzelak et al., 2015; Varga and Kytöviita, 2016). This is because seedlings can be more quickly and efficiently colonized by MNs than by soil resting propagules (Nara, 2006a,b). Besides, seedlings that are recruited into existing MNs gain rapid access to soil resources, and possibly also to carbon derived from other plants connected to the network, usually increasing their chances for establishment and growth (Varga and Kytöviita, 2016). Therefore, MNs integrate multiple plant and fungal species that interact with each other, comprising a complex adaptive social network and influencing the survival, growth, competitive ability, and behavior of the plants and fungi linked to the network (Nara, 2006a; Gorzelak et al., 2015).

Arbuscular mycorrhiza (AM) and ectomycorrhiza (EcM) are the ecologically most important mycorrhizal types. The former is present in 72% of vascular plant species, while the latter can be found in a relatively smaller number of woody species (2%; Smith and Read, 2008; Brundrett and Tedersoo, 2018). EcM-forming plants nevertheless dominate vast areas worldwide, including areas having economic value as the main producers of timber (Smith and Read, 2008; Brundrett and Tedersoo, 2018). AM fungi (AMF) and EcM fungi (EcMF) have different morphologies, growth patterns, and mechanisms for capturing nutrients, and they compete with each other using different strategies (Peay, 2016; Tedersoo and Bahram, 2019). Despite their playing major roles in inorganic P and N uptake, AMF have rather low capacity to release nutrients from sorbed inorganic or organic forms. By contrast, EcMF are able to break down complex organic substrates so that EcM plant species have better access to organic pools of nutrients as compared to AM plants (Lambers et al., 2008; Smith and Read, 2008; Peay, 2016; Montesinos-Navarro et al., 2018). As a result, the AM symbiosis tends to be more abundant in early successional soils, while EcM plants start to proliferate and dominate with the accumulation of soil organic matter (Read, 1991; Lambers et al., 2008; Piotrowski et al., 2008; Peay, 2016).

Succession naturally occurs in environments where new substrates are deposited, such as glacier forefronts, floodplains, lava beds, tephra deposits, or spoil banks formed after mining activities (Allen et al., 2005; Piotrowski et al., 2008; Frouz et al., 2014, 2016; Moguilevsky et al., 2018). These spoil banks are composed of infertile material and characterized by adverse abiotic conditions, such as low nutrient content, high vulnerability to erosion, low drainage ability, and sparse biological activity (Püschel et al., 2007a; Prach et al., 2013; Frouz et al., 2014). In this context, both AM and EcM hosts as well as their associated fungi compete for aboveground (light) and belowground resources (nutrients, water; Haskins and Gehring, 2004; McHugh and Gehring, 2006; Mudrák et al., 2016). It is usually assumed that there is a predictable sequence of mycorrhizal types during primary succession and subsequent ecosystem development, starting with plant species having no or low dependence on mycorrhiza, which are later replaced by AM forbs and grasses, followed by EcM trees with an AM understory (Janos, 1980; Allen et al., 2005; Lambers et al., 2008; Prach et al., 2013; Rydlová et al., 2014; García de León et al., 2016). This is also characteristic for ecological succession on spoil banks (Prach et al., 2013; Rydlová et al., 2014). Therefore, the ecosystems developing on spoil banks constitute ideal systems for studying interactions between plants and fungi forming different mycorrhizal types.

Coexistence of AM and EcM has been investigated in the roots of dual hosts (Johnson et al., 1997; Jones et al., 1998; Hoeksema et al., 2010; Cosme et al., 2018; Teste et al., 2020), but little is known about the interactions of the two mycorrhizal types in soils and the existing evidence is fragmented and sometimes conflicting. For example, it has been observed that EcM hosts can negatively affect the biomass and the occurrence of AMF in understory herbaceous plants as a result of belowground competition (Becklin and Galen, 2009; Becklin et al., 2012; Mudrák et al., 2016). Moreover, growth and mycorrhizal colonization of EcM tree species also can be significantly reduced as a consequence of belowground competition with AM shrubs (Haskins and Gehring, 2004; McHugh and Gehring, 2006). Knoblochova et al. (2017) demonstrated that the coexistence of AM and EcM host species on spoil banks significantly affects their root-associated fungal communities, the effect of the EcM host on the AM plant being distinctly more pronounced.

The main objective of this study was therefore to address the interactions of AM and EcM in controlled experimental conditions, which enable simultaneous evaluation of AMF and EcMF communities in soil and of their influence on the establishment and early development of hosts’ seedlings. We approached this objective by carrying out a greenhouse microcosm experiment using *Betula pendula* and *Hieracium caespitosum* as EcM and AM hosts, respectively. These are two species typically coexisting during early stages of vegetation development (Rydlová et al., 2016). We hypothesized that (1) there would be a reciprocal antagonism between the two types of mycorrhizal fungi such that the abundance, diversity and infectivity of each EcMF and AMF would be negatively affected by the presence of the other’s mycorrhizal host; (2) seedling would grow better in the presence of MNs corresponding to their own mycorrhizal type, while their growth would be reduced by the MNs of the other mycorrhizal host.

## Materials and Methods

### Site Description and Soil Collection

The soil for the greenhouse experiment was collected from an approximately 25-year-old site of a coal mining spoil bank wherein the transition of vegetation from the dominance of AM hosts to EcM hosts was ongoing (50°14′32″ N, 12°40′30′′ E, northwestern Bohemia, Czech Republic). Environmental characteristics of this site were previously described by Rydlová et al. (2016) and Knoblochova et al. (2017). Briefly, during early succession this site was colonized by AM-forming grasses and herbs, mainly *Calamagrostis epigejos*, *H. caespitosum*, *Tussilago farfara*, *Daucus carota*, and *Centaurea stoebe.* At the same time, EcM trees started to appear, in particular *Salix caprea* and *B. pendula.* At the time of sampling, there was a dense understory of AM hosts but EcM trees had begun visibly to dominate the site. Soil was collected from six sampling points (to depth of 0–20 cm), homogenized (while removing large root segments and non-weathered pieces of parent substrate), air-dried, and then stored in a cold and dark place until further use (approximately 3 months).

### Experimental Design

*Betula pendula* and *H. caespitosum* were selected as model species for EcM and AM hosts, respectively, because they are abundant and naturally coexist on the 25-year-old coal mine spoil bank site. For performing the experiment, plastic rhizoboxes (18 × 9 × 16 cm) separated into three equal compartments (6 × 9 × 16 cm) by nylon mesh with 42 μm pore size were used. Hyphae but not roots were able to spread between the compartments (Janoušková et al., 2011). The substrate used for filling the two lateral compartments was a mixture (1:1 v/v) of the non-sterile spoil bank soil and autoclaved (30 min at 121°C) zeolite. The middle compartment was filled with the same substrate, but it had been sterilized by γ-irradiation (25 kGy). Seeds of *H. caespitosum* were collected on different sites on the spoil banks, while seeds of *B. pendula* were purchased from a seed producer (Lesy České Republiky, Týniště nad Orlicí, Czech Republic). All seeds were surface-sterilized with a 10% solution of sodium hypochlorite for 5 min. Seeds were germinated and seedlings pre-grown under semi-sterile conditions in transparent plastic boxes containing autoclaved river sand.

**Figure 1:**
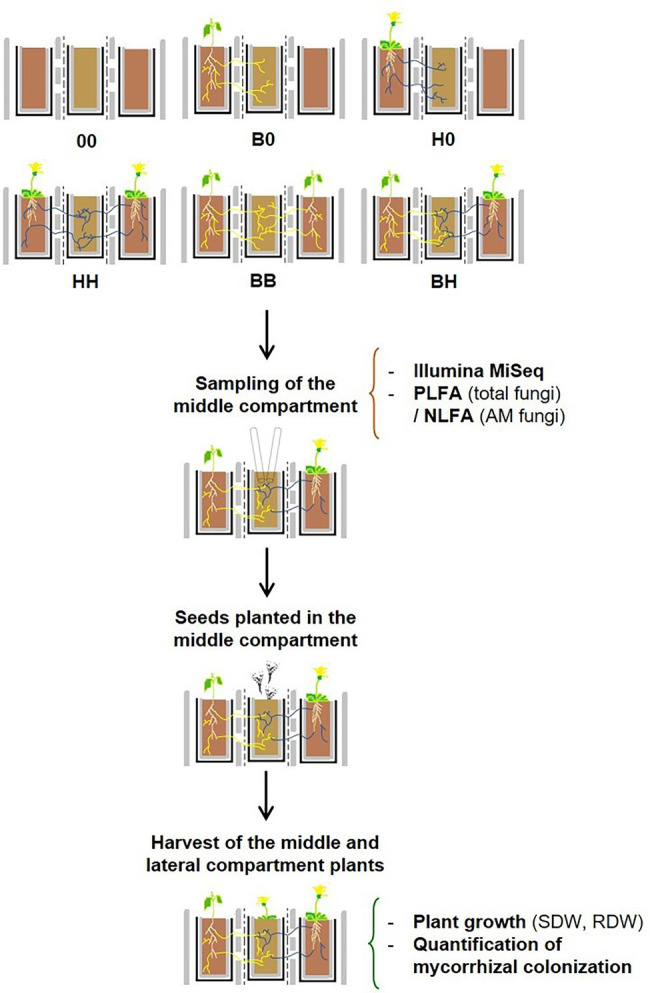
Diagram of the experimental design. The two lateral compartments of three-compartment rhizoboxes were left unplanted or planted with *Betula pendula* hosting ectomycorrhizal fungi (yellow lines) and/or with *Hieracium caespitosum* hosting arbuscular mycorrhizal fungi (blue lines) according to six different treatments (Stage I, *n* = 16). Seedlings of either *B. pendula* or *H. caespitosum* were planted into the middle compartments of each treatment after 5 months, thus resulting in 12 treatments in Stage II (*n* = 8). Treatments: 00 = without plants in lateral compartments, B0 = *B. pendula* in only one lateral compartment, BB = *B. pendula* in both lateral compartments, BH = *B. pendula* and *H. caespitosum* in lateral compartments (interaction treatment), H0 = *H. caespitosum* in only one lateral compartment, HH = *H. caespitosum* in both lateral compartments. NLFA = neutral lipid fatty acid analysis, PLFA = phospholipid fatty acid analysis, RDW = root dry weight, SDW = shoot dry weight.

The experiment consisted of two stages (Figure 1). In Stage I (conditioning phase), 7 weeks old seedlings of AM and/or EcM hosts were transplanted into the lateral compartments (hereinafter referred to as “large plants”) to establish the following treatments: (a) lateral compartments without plants (00, the lateral rhizoboxes contained bare soil throughout the experiment), (b) only one lateral compartment occupied by *B. pendula* (B0) or *H. caespitosum* (H0), or (c) both lateral compartments planted with the same plant species (BB and HH) or with a combination of both (BH, hereinafter referred to also as the “interaction treatment”). To avoid airborne contamination, the middle compartment was covered with an aluminum foil until seeds were sown into it. Sixteen replicates were established per treatment (00, B0, H0, HH, BB, and BH), and thus there were 96 rhizoboxes in total. The plants were cultivated for 5 months in a temperature-controlled greenhouse (18–28°C) with supplemental lighting (400 W metal halide bulbs) and watered with deionized water according to need. After 5 months, soil samples were collected from the middle compartments to describe the fungal communities. Two subsamples were taken from diagonal corners of the middle compartment using a sterile laboratory spoon to the depth of about 4–5 cm. The two subsamples were pooled (ca 10 g of soil fresh weight in total), homogenized, then separated into two parts: one of them for phospholipid fatty acid (PLFA) and neutral lipid fatty acid (NLFA) analyses and the second for DNA-based characterization of the soil fungal communities (Figure 1).

For Stage II, one-half of the rhizoboxes of each treatment was planted with 20 seeds of *B. pendula* (EcM host) and the second half with 25 seeds of *H. caespitosum* (AM host). Seeds were evenly placed on the soil surface of the middle compartments and watered. To prevent desiccation, the middle compartments were covered for a week with transparent foil lids that enable gas flow and prevent mold growth. In this second part of the experiment, there were eight replicates per plant species and treatment. Seed germination was recorded for 2 weeks and did not differ between the experimental treatments (data not shown). The two most distant and healthy-looking seedlings present in each of the middle compartments were retained and extra seedlings were removed by cutting. After 4 months under the same greenhouse conditions, the seedlings from the middle compartments were harvested (Figure 1).

### Estimation of Fungal Biomass

Analyses of NLFA and PLFA were performed for each soil sample in order to estimate the fungal biomasses. The former enables quantification of AMF biomass (Olsson, 1999; Sharma and Buyer, 2015), while the latter is a better estimator of the biomass of the other fungal groups present in the soil, including EcMF, saprophytic (SaprF), and pathogenic fungi (PathF). Soil samples were extracted using a chloroform–methanol–phosphate buffer mixture (1:2:0.8) as detailed by Frouz et al. (2016). Briefly, the extracted lipids were separated using solid-phase extraction cartridges (LiChrolut Si 60, Merck). The samples were eluted in three fractions containing neutral lipids, glycolipids, and phospholipids with 2 ml of chloroform, 6 ml of acetone, and 2 ml of methanol, respectively (Oravecz et al., 2004). The first and third fractions were subjected to mild alkaline methanolysis (Šnajdr et al., 2008). The free methyl esters of NLFA and PLFA were analyzed by gas chromatography–mass spectrometry (450-GC, 240-MS ion trap detector, Varian, Walnut Creek, CA, United States). The GC instrument was equipped with a split/splitless injector, and a DB-5MS column was used for separation (60 m, 0.25 mm i.d., 0.25 mm film thickness) according to the programs described by Frouz et al. (2016). Mass spectra were recorded at 1 scan s^−1^ under electron impact at 70 eV, mass range 50–350 amu. Methylated fatty acids were identified according to their mass spectra and by using a mixture of chemical standards from Sigma-Aldrich (St. Louis, MO, United States) and Matreya LLC (Pleasant Gap, PA, United States). Fungal biomass in the PLFA fraction was quantified based on 18:2ω6.9 concentration (Stella et al., 2015) while the biomass of AMF was estimated using 16:1ω5 concentration in the NLFA fraction (Bååth, 2003; Frouz et al., 2016).

### Characterization of Fungal Communities

The composition of fungal communities was assessed in the soil samples taken at the end of the Stage I (Figure 1). DNA was extracted from 2 g of soil per sample using the PowerSoil^®^ DNA Isolation Kit (Mobio) according to the manufacturer’s instructions. Polymerase chain reaction (PCR) was performed using the primer pair gITS7ngs (5′-GTGARTGTGARTCATCRARTYTTTG-3′; Ihrmark et al., 2012) and ITS4 (5′-TCCTCCGCTTATTGATATGC-3′; White et al., 1990) to amplify the ITS2 region. Both forward and reverse primers were tagged by molecular identifiers containing 10–11 bases. Three separate PCR reactions were performed for each sample to reduce PCR bias. The PCR mix consisted of 2.5 μl of 10× Taq buffer, 1 μl of dNTPs mix (10 mM), 2 μl of MgCl2, 1 μl of each primer (5 μM), 0.2 μl of Taq polymerase (Thermo Scientific), 1 μl of bovine serum albumin, 2 μl of 10 times diluted DNA, and 15.8 μl of ddH2O in a total volume of 26.5 μl. The cycling conditions were 4 min at 94°C, followed by 35 cycles of 30 s at 94°C, 30 s at 49°C, and 40 s at 72°C, then a final extension of 10 min at 72°C. Technical amplicon replicates of suitable PCR products were mixed together per each sample and then purified using the Qiaquick PCR purification kit (Qiagen). The DNA concentration was then measured using Qubit (Life Technologies) and all the PCR products were equimolarly pooled and then sequenced using the Illumina MiSeq system at GATC Biotech (Cologne, Germany).

SEED pipeline v 2.1.05 (Vetrovský et al., 2018) was used for filtering and quality check of ~2,200,000 reads obtained from Illumina MiSeq. The reads were merged into paired-end sequences with at least 20 bp overlap and maximum difference 15%. All sequences shorter than 40 bp and average base quality scores lower than 38 were removed from the data set. Sequences without primers and identifiers as well as those with mismatched identifiers also were removed. The remaining sequences were sorted into samples according to the molecular identifier sequences. The fungal ITS2 was extracted using ITSx (Bengtsson-Palme et al., 2013) and the ITS2 sequences were clustered by implementing UPARSE in USEARCH on the 97% similarity level. Chimeric sequences together with singletons and doubletons were removed from the data set. From each cluster (altogether 840 OTUs), the most abundant sequence was selected for BLAST search against the NCBI GenBank (altogether 840 OTUs, Supplementary Table S1). Operational taxonomic units (OTUs) were assigned to fungal ecological guilds using FUNGuild v 1.0 (Nguyen et al., 2016) with subsequent manual corrections (Supplementary Table S1). To avoid the effect of unequal read numbers per sample in linear statistical analyses, 1,190 reads per sample were randomly subsampled in R (Kolaříková et al., 2017).

### Determining Plant Biomass and Mycorrhizal Root Colonization

Shoot (SDW) and root dry weight (RDW) were determined in all plants by drying at 60°C to constant weight. For analyzing mycorrhizal colonization, root samples were stained with 0.05% Trypan blue in lactoglycerol (Koske and Gemma, 1989). Thirty root segments ca 1.5 cm in length were observed under a compound microscope (Olympus IX 51) at 200× magnification. AM colonization was evaluated according to Trouvelot et al. (1986), and three colonization parameters were estimated using the program “Mycocalc”:^1^ frequency of mycorrhizal colonization (AM.F), abundance of arbuscules (AM.A), and abundance of vesicles (AM.V; Janoušková et al., 2011; Rydlová et al., 2016). For *B. pendula*, the presence of EcM structures (hyphal mantle and Hartig net) was scored in 100 microscopic fields per sample to calculate colonization frequency (EcM.F).

### Statistical Analyses

To test the effect of the experimental treatments on the concentrations of fungal PLFA and NLFA in soil, AM and EcM colonization rates (AM.F, AM.M, AM.A, AM.V, EcM.F), and plant growth (SDW, RDW), we used the IBM SPSS software v. 23.0 (IBM Corp.). The data sets were first checked for normality (Shapiro–Wilk test) and homogeneity of variance (Levene’s test). Non-parametric Kruskal–Wallis ANOVA followed by Nemenyi post-hoc tests were performed for variables that showed non-normal distribution even after transformation (i.e., PLFA and NFLA, numbers of sequences and OTUs per fungal guild, mycorrhizal colonization, and seedling growth). Additionally, mycorrhizal colonization rates and growth of large plants were analyzed using two-way ANOVAs followed by Holm–Sidak pairwise multiple comparison tests (Factor 1 = Treatment: 00, B0, BB, BH, H0, and HH; Factor 2 = plant species sown in the middle compartment: *B. pendula* and *H. caespitosum*). Pearson correlations were calculated to investigate the association between seedling growth (SDW, RDW) and mycorrhizal colonization.

Fungal communities were standardized by Hellinger transformation, and Bray–Curtis dissimilarity was used to construct a fungal community dissimilarity matrix. PERMANOVA using the adonis function of the “vegan” package in R was applied to address the effect of the experimental treatments on the fungal community composition, followed by pairwise PERMANOVA with 99,999 permutations to determine specific differences between treatments. Bonferroni correction was used to calculate the corrected values of *p* from those determined by the pairwise PERMANOVA analyses. The heatmap function in R v. 3.5.1 (R Core Team, 2018) was used for evaluating similarities between the fungal communities present in the soils of the different treatments.

## Results

### Effect of AM and EcM Plants on the Soil Fungal Communities

**Figure 2:**
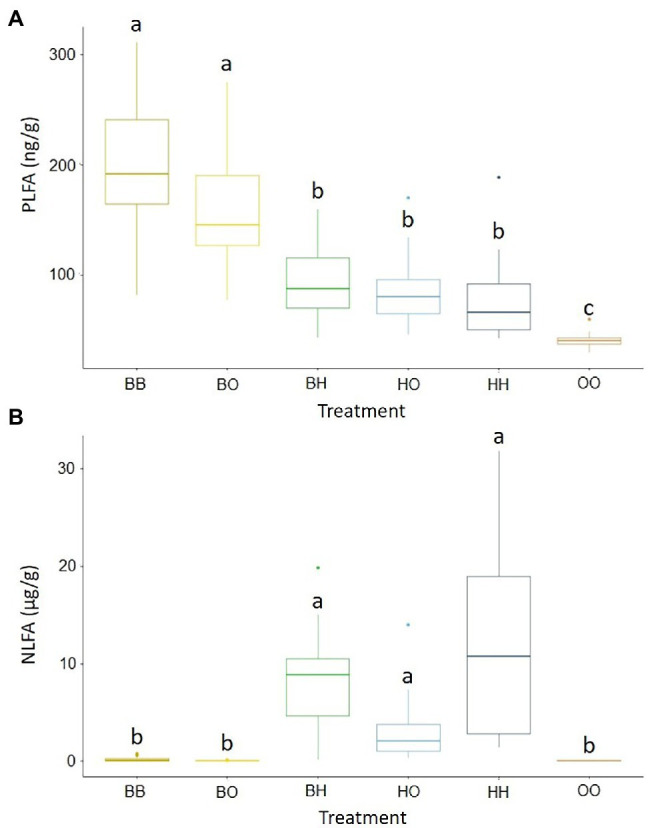
Concentrations of phospholipid fatty acid analysis (PLFA) 18:2ω6.9 **(A)**, an estimator of total fungal biomass, and neutral lipid fatty acid analysis (NLFA) 16:1ω5 **(B)**, an estimator of arbuscular mycorrhizal fungal biomass, in the middle compartments of the experimental treatments. Different letters above the boxplots indicate significant differences between treatments. The midline of the boxplots is the median, the upper and lower limits of the box the third and first quartile, respectively. Whiskers extend to 1.5 times the interquartile range from the top (bottom) of the box (*n* = 16). Treatments: 00 = without plants in lateral compartments, B0 = *B. pendula* in only one lateral compartment, BB = *B. pendula* in both lateral compartments, BH = *B. pendula* and *H. caespitosum* in lateral compartments (interaction treatment), H0 = *H. caespitosum* in only one lateral compartment, HH = *H. caespitosum* in both lateral compartments.

Fungal biomass (as PLFA 18:2ω6.9) in soil was significantly greater in treatments with only *B. pendula* (BB, B0) than in treatments where *H. caespitosum* was present in at least one of the compartments (BH, H0, HH). The lowest values were recorded in the treatment without any plants (Figure 2A), thus indicating that most of the PLFA 18:2ω6.9 content corresponded to EcMF rather than to SaprF or PathF. On the other hand, AMF biomass (as NLFA 16:1ω5) was significantly greater in all the treatments with *H. caespitosum* (BH, H0, HH) than in the others (Figure 2B). These results show that the interaction between *B. pendula* and *H. caespitosum* had a more negative effect on the overall fungal biomass (mostly comprised of EcMF) than on AMF biomass.

**Figure 3:**
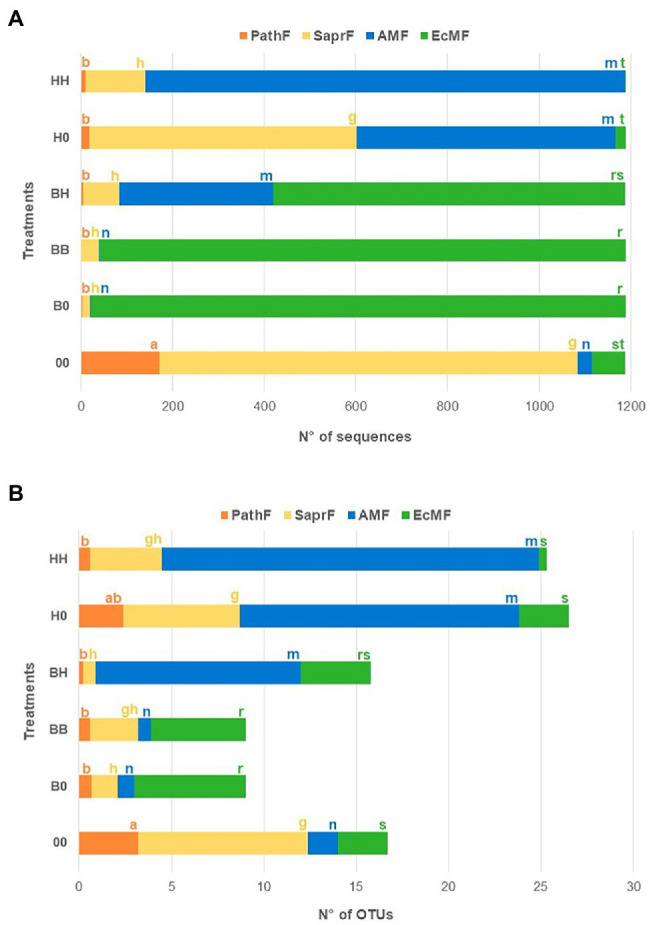
Relative abundance **(A)** (mean number of sequences) and richness **(B)** (mean number of operational taxonomic units -OTUs) of each fungal guild in the middle compartments of the experimental treatments. Fungal guilds: EcMF = ectomycorrhizal fungi, AMF = arbuscular mycorrhizal fungi, SaprF = saprotrophic fungi, PathF = pathogenic fungi. For each fungal guild significant differences between treatments (*n* = 16) are indicated with different letters (a–b = PathF, g–h = SaprF, m–n = AMF, r–s = EcMF). Treatments: 00 = without plants in lateral compartments, B0 = *B. pendula* in only one lateral compartment, BB = *B. pendula* in both lateral compartments, BH = *B. pendula* and *H. caespitosum* in lateral compartments (interaction treatment), H0 = *H. caespitosum* in only one lateral compartment, HH = *H. caespitosum* in both lateral compartments.

The relative abundance of EcMF (measured as number of sequences) was significantly higher in treatments having *B. pendula* in at least one of the compartments (BB, B0, BH) than in treatments with only *H. caespitosum* (H0, HH; Figure 3A). The highest EcMF taxa richness (measured as number of OTUs) was recorded in treatments with only *B. pendula* (BB, B0) and the lowest in those within which the EcM host was not present (H0, HH, 00). Intermediate values of EcMF taxa richness were determined in the interaction treatment (BH; Figure 3B). On the other hand, the relative abundance and richness of AMF were significantly higher in all treatments with *H. caespitosum*, including the interaction treatment (H0, HH, BH), than in treatments without the AM host (B0, BB, 00). The highest relative abundance of PathF and SaprF was recorded in the treatment without any plants in the lateral compartments (00; Figure 3).

**Figure 4:**
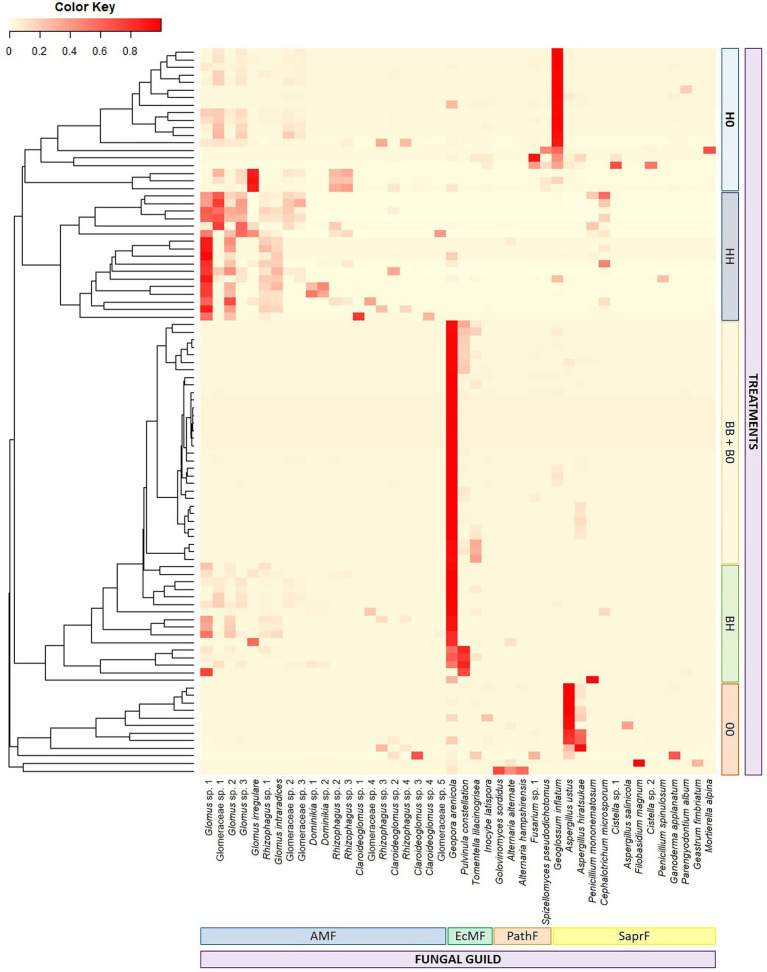
Heatmap displaying composition of fungal communities in middle compartments of the experimental treatments. Due to visualization limitations, operational taxonomic units with less than 10% as their maximum relative abundance were not considered. Fungal guilds: AMF = arbuscular mycorrhizal fungi, EcMF = ectomycorrhizal fungi, PathF = pathogenic fungi, SaprF = saprotrophic fungi (*n* = 16). Treatments: 00 = without plants in lateral compartments, B0 = *B. pendula* in only one lateral compartment, BB = *B. pendula* in both lateral compartments, BH = *B. pendula* and *H. caespitosum* in lateral compartments (interaction treatment), H0 = *H. caespitosum* in only one lateral compartment, HH = *H. caespitosum* in both lateral compartments.

Fungal community composition differed significantly among almost all treatments (Figure 4; Supplementary Table S1), the only exception being those treatments where *B. pendula* was the only cultivated plant species (BB and B0). Fungal communities from BB and B0 treatments were similar and characterized by high abundance of EcMF, especially of *Geopora arenicola*. The BH treatment was characterized by a combination of EcMF and AMF that led to a significantly different composition as compared to the other treatments. The large relative share of AMF in samples corresponding to treatments with only *H. caespitosum* (H0 and HH) distinguished these treatments from the others, and the presence of SaprF differentiated the H0 treatment from HH. Samples without any cultivated plants (00) showed high relative abundance of SaprF and PathF as well as low occurrence of the mycorrhizal guilds (Figure 4). When PERMANOVA was performed separately for analyzing AMF communities in the treatments with *H. caespitosum* (H0, HH, and BH) and EcMF in those with *B. pendula* (B0, BB, and BH), the effect of the presence of the other mycorrhizal host in the cultivation system differed between the two mycorrhizal fungal guilds. There were no differences in the composition of AMF communities among any of those treatments having *H. caespitosum* in at least one of the lateral compartments. By contrast, the EcMF community composition was similar in both treatments with only *B. pendula*, but it differed significantly when this species was cocultivated with the AM host (BH treatment; Supplementary Table S1).

### Plant Growth and Mycorrhizal Root Colonization

**Figure 5:**
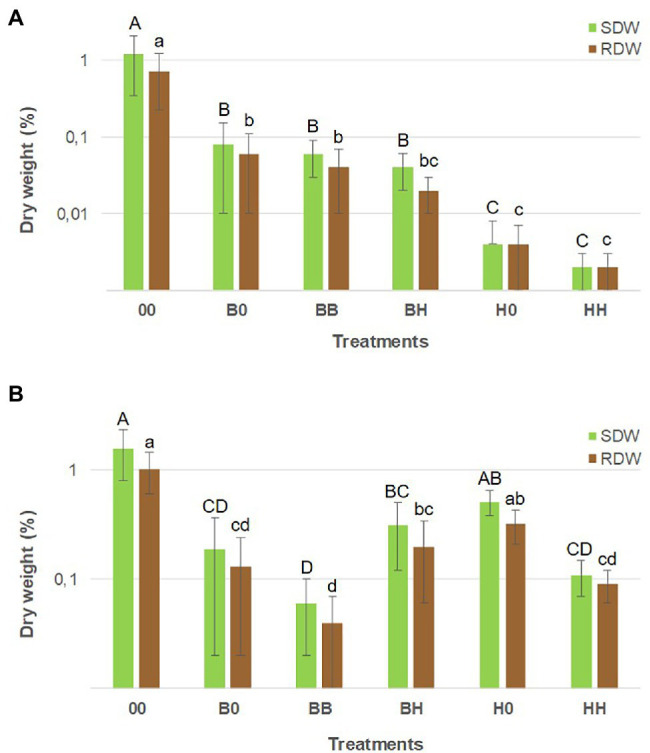
Biomass of *B. pendula* **(A)** and *H. caespitosum* **(B)** seedlings in middle compartment of each treatment. A logarithmic scale is used on the y-axis. For each variable, significant differences between treatments (*n* = 8) are indicated with different letters [uppercase for shoot dry weight (SDW) and lowercase for root dry weight (RDW)]. Treatments: 00 = without plants in lateral compartments, B0 = *B. pendula* in only one lateral compartment, BB = *B. pendula* in both lateral compartments, BH = *B. pendula* and *H. caespitosum* in lateral compartments (interaction treatment), H0 = *H. caespitosum* in only one lateral compartment, HH = *H. caespitosum* in both lateral compartments.

**Figure 6:**
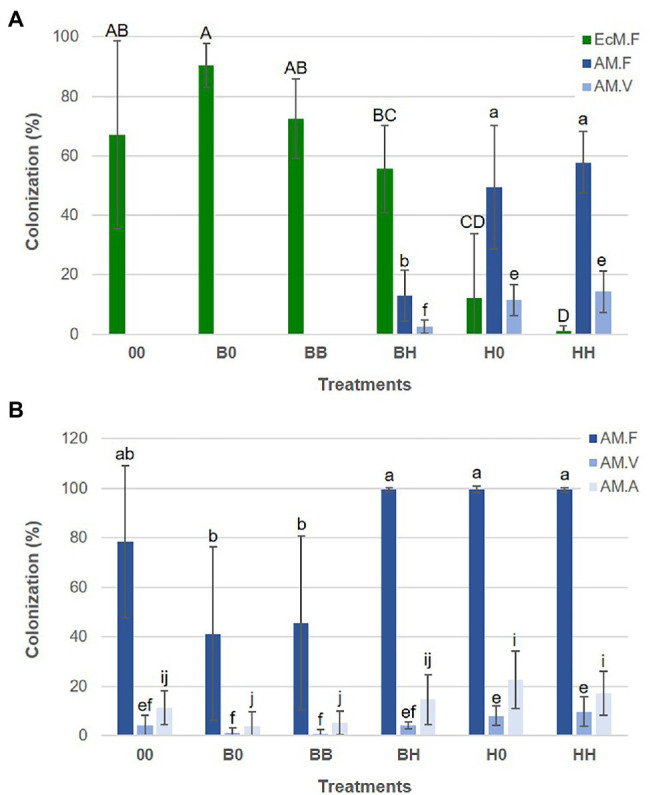
Mycorrhizal colonization of *B. pendula* **(A)** and *H. caespitosum* **(B)** seedlings in each treatment. EcM.F = frequency of EcM colonization, AM.F = frequency of AM colonization, AM.V = abundance of vesicles, AM.A = abundance of arbuscules. For each variable, significant differences between treatments (*n* = 8) are indicated with different letters (uppercase for EcM.F and lowercase for AM.F [a-b], AM.V [e-f] and AM.A [i-j]). Treatments: 00 = without plants in lateral compartments, B0 = *B. pendula* in only one lateral compartment, BB = *B. pendula* in both lateral compartments, BH = *B. pendula* and *H. caespitosum* in lateral compartments (interaction treatment), H0 = *H. caespitosum* in only one lateral compartment, HH = *H. caespitosum* in both lateral compartments.

Growth and mycorrhizal colonization of *H. caespitosum* and *B. pendula* seedlings varied widely among the treatments. For both plant species, the largest seedlings were those growing in the treatment without plants in the lateral compartments (Figure 5). Growth and EcM colonization of *B. pendula* seedlings were significantly reduced in treatments where *H. caespitosum* was the only plant species (H0, HH) as compared to treatments with *B. pendula* only (B0, BB). While biomass of *B. pendula* seedlings was not significantly decreased in the interaction treatment (BH) as compared to treatments where *B. pendula* was the only cultivated plant (B0, BB; Figure 5), EcM colonization was significantly less in this treatment than in B0 (Figure 6). In treatments that included *H. caespitosum* in at least one of the lateral compartments (BH, 0H, HH), *B. pendula* seedlings were also colonized by AMF. However, arbuscules were never detected in *B. pendula* roots in our experiment. The AM colonization of *B. pendula* was significantly less in the interaction treatment (BH) as compared to HH and H0 (Figure 6).

Biomass of the *H. caespitosum* seedlings in treatment H0 was significantly larger than that in B0, HH, and BB, while those growing in the interaction treatment (BH) had intermediate SDW and RDW values (Figure 5). Arbuscular mycorrhizal colonization was significantly greater in all those treatments having *H. caespitosum* in at least one of the lateral compartments (H0, HH, BH) than in those with only *B. pendula* (B0, BB). Arbuscular mycorrhizal colonization of *H. caespitosum* in the treatment without neighboring plants (00) showed intermediate values (Figure 6).

The tendencies observed for the seedlings were consistent with the results obtained for the large plants (Supplementary Table S1). Plant biomass and EcM colonization were significantly less in *B. pendula* specimens from the interaction treatment (BH) than in conspecific treatments (BB, B0), while *H. caespitosum* was not negatively affected by the presence of the other mycorrhizal host (BH treatment).

In treatments with plants in the lateral compartments (excluding the 00 treatment), it was observed for both plant species that when the seedlings were grown together with the other mycorrhizal host, root colonization values (AM.F for *H. caespitosum* or EcM.F for *B. pendula*) were positively and significantly correlated with the seedlings’ growth (Table 1; Supplementary Table S1). By contrast, in absence of the other mycorrhizal host, growth was unaffected by mycorrhizal colonization (Table 1). In *B. pendula* it was also noticed that seedling growth (Table 1) and EcM.F (*ρ* = −0.906, *p* < 0.001) were significantly and negatively correlated with AM colonization values (Supplementary Table S1).

**Table 1:** Correlations between growth parameters (shoot dry weight, root dry weight) and mycorrhizal colonization (AM.F, EcM.F) in *B. pendula* and *H. caespitosum* seedlings, either in presence (+) or absence (−) of the opposite mycorrhizal host in the cultivation system.

	*B. pendula*		*H. caespitosum*
	*H (+)^a^*		*H (−)^b^*		*B (+)^c^*	*B (−)^d^*
	AM.F	EcM.F	AM.F	EcM.F	AM.F	AM.F
SDW	−0.73^***^	0.72^***^	–	0.12^ns^	0.62^**^	0.02^ns^
RDW	−0.70^***^	0.71^***^	–	0.12^ns^	0.56^**^	-0.57^ns^

Correlation is significant at ^***^p < 0.001, ^**^p < 0.01, ns = non-significant. H = *H. caespitosum*; B = *B. pendula*; ^a^ = treatments HH, H0, BH; ^b^ = treatments BB, B0, 00; ^c^ = treatments BB, B0, BH; ^d^ = treatments HH, H0, 00. SDW = shoot dry weight, RDW = root dry weight, AM.F = frequency of AM colonization, EcM.F = frequency of EcM colonization.

## Discussion

As expected, the mycorrhizal type of the large plants affected the community composition of soil fungi as well as mycorrhizal colonization and growth of the seedlings. Nevertheless, and in contrast to our hypothesis, the coexistence of both plant species and their mycorrhizal fungi did not lead to reciprocal antagonism, since the abundance and composition of EcMF in the soil and the growth of the EcM seedlings were negatively affected by the AM host and associated AMF but not vice versa. To our best knowledge, this is the first time that the interaction between EcMF and AMF and its effect on seedlings establishment and growth have been addressed under controlled conditions. Despite this type of approach simplifies the influence of diverse environmental conditions on the study system, it is useful for reducing the complex impact of different, potentially interacting environmental factors on this dynamic system. Consequently, we were able to explore how different mycorrhizal types interact with each other, shedding some light on interactions which happen in natural conditions. Contrasting our results with those of previous studies from more complex settings and carried out under natural conditions, we deduce and discuss factors that influence the specific outcome of these interactions.

### Soil Fungal Communities and Mycorrhizal Colonization

The asymmetric response of the two mycorrhizal fungal guilds to the presence of the other host (and associated fungi) was evident at the level of the fungal biomass in soil, root colonization, and community composition. It suggests that, in our selected plant species and experimental conditions, AMF were less affected by competition with EcMF than vice versa. Asymmetric response of EcMF and AMF abundance to the presence of the other mycorrhizal type has been described for other plant species combinations. For example, willows indirectly reduced AMF abundance in roots of herbaceous plants *via* feedbacks with leaf litter and EcMF (Becklin et al., 2012; Mudrák et al., 2016). Similarly, Knoblochova et al. (2017) determined that presence of the ectomycorrhizal host *S. caprea* negatively affected the abundance of AM fungi not only in soil but also in *C. epigejos* roots, while *C. epigejos* caused no suppression of EcMF. In contrast, and similarly to what was observed in our study, McHugh and Gehring (2006) established that belowground competition with AM shrubs negatively impacted the abundance of EcM in roots of pinyon pines (*Pinus edulis*). When different mycorrhizal guilds occur in a nutrient-limited environment they have to acquire and provide the same resources to their host plants, thus resulting in this type of asymmetric effects and antagonistic plant–plant interactions (Montesinos-Navarro et al., 2018). The outcome of the interaction may then be related to the different nutrient acquisition strategies of these mycorrhizal guilds. To use the language of Lambers et al. (2008), the “scavenging strategy” of AMF is more efficient in early successional soils while the contrasting “mining” strategy of EcMF is superior in utilizing nutrients in later successional soils, where nutrients are usually bound in organic compounds (Read, 1991; Lindahl and Tunlid, 2015; Peay, 2016). Because we studied a 20-year-old coal mine spoil bank in which organic matter was absent or very scarce (Rydlová et al., 2016), it is likely that AMF were better competitors for the available resources than were EcMF, thus outcompeting them in the interaction treatment. Our results are also consistent with the “mutualistic niche concept” developed by Peay (2016), who stated that AM hosts and fungi tend to exclude EcM plants and fungi when organic N supply is low with respect to inorganic N, as it was in the spoil bank soil.

In contrast to our results, however, preceding studies in the same successional system determined that when an AM host (*C. epigejos*) coexisted with an EcM host (*S. caprea*), the latter affected negatively the abundance and richness of AMF as well as altered their community composition, while the impact of the AM host on EcMF communities was mostly not significant (Mudrák et al., 2016; Knoblochova et al., 2017). There are several plausible explanations for this discrepancy. It can be a matter of developmental stage and/or environmental conditions (Lodge and Wentworth, 1990), because adult plants from natural ecosystems were examined in those studies while young plants and seedlings cultivated under controlled conditions were analyzed in our study. Another probable reason is that different plant species were considered in each of these works. Detrimental effects of EcM plants and associated root fungi on AM hosts and AMF communities have been largely proven for *Salix* (Becklin and Galen, 2009; Becklin et al., 2012; Mudrák and Frouz, 2012; Mudrák et al., 2016; Knoblochova et al., 2017), but the opposite situation has been reported for *P. edulis* (Haskins and Gehring, 2004; McHugh and Gehring, 2006). This is the first time that the interaction between EcM and AM was studied using *B. pendula* as the EcM model plant. A factor that cannot be neglected is the allelopathy associated with some plant species but not with others. For instance, it is known that *S. caprea* produces allelopathic compounds (Mudrák and Frouz, 2012), and, despite that their effects on AMF are not well known, it has been hypothesized several times that they negatively influence the abundance and diversity of these fungi in soil (Becklin and Galen, 2009; Becklin et al., 2012; Mudrák and Frouz, 2012; Mudrák et al., 2016; Knoblochova et al., 2017). In contrast, allelopathic effects of *Betula* on soil fungi have not been described (Michelsen et al., 1995; Huang et al., 2016).

For seedlings of both plant species, mycorrhizal colonization was lowest when the other mycorrhizal host was the only plant in the lateral compartments. This is probably a consequence of missing MNs and the necessity to establish the colonization from spores remaining in the soil of the lateral compartments. Interestingly, the need to mention that mycorrhizal colonization from spores was also relevant for seedlings growing without any neighboring plants (treatment 00). In this case, however, mycorrhizal colonization of *H. caespitosum* and *B. pendula* seedlings was as great as in the treatments with conspecific neighboring plants. This suggests that the presence of MNs formed by fungi of a certain mycorrhizal type suppresses development of the other mycorrhizal type, and, interestingly, this effect was symmetric between the two fungal groups in our experiment. This is in agreement with other studies that also have documented how different plant species influence the abundance of mycorrhizal fungi in the soil and roots of neighboring plants through the production of inhibitory compounds or competitive interactions (Nilsson et al., 1993; McHugh and Gehring, 2006), which probably are mediated by associated mycorrhizal networks. Janos et al. (2013) even demonstrated that AM networks can be actively antagonistic to potential EcM hosts.

It is noteworthy that *B. pendula* plants were colonized by AM fungal hyphae and vesicles only when they coexisted with *H. caespitosum* and had contact with MNs radiating from the AM host, but not from resting propagules in the soil. This phenomenon has been described previously for other plant species that usually do not form AM (Püschel et al., 2007a; Janoušková et al., 2011; Cosme et al., 2018; Teste et al., 2020). Cosme et al. (2018) proposed to classify as “rudimentary AM phenotypes” those plant species that suppress or have lost their ability to form prominent AM phenotypes but under specific circumstances can harbor some symbiotic structures in their roots. These plant species might therefore have sufficient genetic tools to activate components of the symbiotic behavior of AMF (Teste et al., 2020). *B. pendula* could be included into this group of rudimentary AM plants. It is also interesting that, despite the comparable abundance and richness of AMF in all the treatments with *H. caespitosum* in at least one of the lateral compartments, AM colonization in *B. pendula* seedlings from the interaction treatment was significantly less than in treatments with only *H. caespitosum*. This showed that presence of the ectomycorrhizal host increased the abundance of EcMF in the soil as well as EcM formation, whose mantle could have restricted the entrance of AMF (Lodge and Wentworth, 1990). Exclusion of AM colonization in usually pure EcM plants may reflect fungal competition rather than plant control inasmuch as EcMF may outcompete AMF using mechanisms, such as mycelial overgrowth or by colonizing roots prior to other competitors (Teste et al., 2020). Another particularity observed in the soil of treatments including *B. pendula* was the dominance of the EcMF *G. arenicola. Geopora* species have been mainly found in *Pinus* spp. (Flores-Rentería et al., 2014 and references within; Shemesh et al., 2019), and occasionally in other tree species (Southworth and Frank, 2011; Long et al., 2016). As far as we know this is the first time it has been registered in association with *B. pendula*. These EcMF species are considered as stress tolerant, since their relative abundance in plant roots increases significantly with drought (Flores-Rentería et al., 2014 and references within), and they can dominate the EcMF spore banks above mountain treelines (Shemesh et al., 2019). The fact that *G. arenicola* was dominant in spoil banks soils agrees with the high tolerance of these species to harsh environmental conditions.

Most of the experiment’s treatments were characterized by a particular assembly of fungi and, in addition to the observed differences regarding mycorrhizal guilds, we also identified shifts in the relative abundance of saprotrophic fungi. In general, relative abundance of saprotrophs was particularly low in treatments with *B. pendula*. This is in agreement with Bödeker et al. (2016), who demonstrated that even though SaprF and EcMF may have overlapping fundamental niches in forests, the latter can restrict saprotrophs mainly through competitive interactions. As these fungal guilds occupy similar vertical positions in the soil profile and target the same litter and soil organic matter substrates, SaprF are usually displaced by EcMF (the phenomenon being known as the “Gadgil effect”). This competition for resources between SaprF and EcMF may be strongest when soils are poorly developed (Fernandez and Kennedy, 2015), as was the soil considered in this study.

Asymmetric responses between different mycorrhizal guilds naturally occur in nature and seem to be independent of plant habit, been possible that EcM tree species suppress herbaceous or shrubby AM species (Becklin et al., 2012; Mudrák et al., 2016; Knoblochova et al., 2017) and vice versa (Haskins and Gehring, 2004; McHugh and Gehring, 2006; this study). Based on the presented information, it becomes evident that the interaction of different mycorrhizal hosts on their fungal partners is context dependent and reliant on multiple biotic and abiotic factors, such as soil properties, the mycorrhizal hosts involved, and neighboring plant species (Moora and Zobel, 1996; Hubert and Gehring, 2008; Gorzelak et al., 2015; Knoblochova et al., 2017). This highlights the importance of studying the interaction between EcM and AM hosts and their fungal partners using different model plants and experimental approaches, including field analyses and cultivation experiments.

### Plant Growth

For both plant species, the largest seedlings were present in the treatment with no neighboring large plant (treatment 00), which corresponds to the early successional life strategy of the studied plant species and their ability to grow rapidly under weak competition. Furthermore, the seedlings may have had access to higher nutrient levels, as the MNs radiating from the large plants previously established in the lateral compartments may have caused nutrient pre-depletion of the soils in the other treatments (Janoušková et al., 2011). In treatments having at least one plant in the lateral compartments the response of the seedlings depended upon the plant species.

The growth of *B. pendula* was significantly reduced when *H. caespitosum* was the only neighboring plant as compared to specimens that were growing together with conspecifics (treatments BB, B0, and BH). Negative effects of AM on EcM hosts have been described previously. For example, Jones et al. (1998) reported that *Eucalyptus coccifera* (dual host) seedlings were smaller and had lower P content when they were colonized by AMF. A question that arises from these observations is whether the negative effects exerted by *H. caespitosum* and its AMF on *B. pendula* seedlings were indirect or direct. Regarding the former case, it is conceivable that *H. caespitosum* and its AMF produced higher nutrient depletion in the middle compartment and/or were better competitors for resources than were *B. pendula* seedlings. The fact that *B. pendula* seedlings were not significantly affected by *H. caespitosum* in the interaction treatment (BH) can be attributed to the greater abundance of EcMF in this treatment. This may counteract the adverse effects exerted by the AM host and associated AMF. It is also possible that AM root colonization reduced EcM colonization and consequently the benefits obtained from this symbiosis. On the other hand, AMF may also have acted directly as “parasitic fungi” (Johnson et al., 1997; Hoeksema et al., 2010). When different plant species connect to existing MNs, a strong asymmetry in the terms of trade may occur (Walder et al., 2012). In our case, it might be that when *B. pendula* seedlings connected to the existing AM networks, they deprived them of significant amounts of carbon and gave little (if any) profit in return (Cosme et al., 2018 and references within), thus directly and negatively affecting the EcM host.

Differences in seedlings growth were also observed for *H. caespitosum*, but the beneficial effects of the conspecific large plants were not as clear as for *B. pendula*. This was probably a consequence of the interaction between different factors conditioning the *H. caespitosum* seedlings’ development, such as AMF inocula and root colonization (significantly lower in treatments with only *B. pendula* in the lateral compartments), nutrient depletion, and availability of MNs. This is in agreement with Püschel et al. (2007b), who suggested that the general growth response of plants to mycorrhizae is probably determined by multiple factors, mainly by soil nutrient content, other plants connected to the MNs, and sharing the costs of network maintenance. For instance, it may have happened that large plants of *H. caespitosum* produced greater nutrient depletion than did *B. pendula*, so that seedlings growing in the treatment with the AM host in both lateral compartments were smaller than those which had this species in only one lateral compartment (H0 and BH). It is also possible that seedlings that become interconnected into AM MNs do not obtain significant amounts of nutrients from the fungus because most of the resources are directed to the dominant carbon source, that is, the larger plants (Moora and Zobel, 1996; Janoušková et al., 2011).

We also observed that when any of the studied species was growing together with the other mycorrhizal host, mycorrhizal colonization was lower but nevertheless positively correlated with the seedlings’ growth. Positive correlations between AM or EcM colonization and plant growth have been widely reported (e.g., Thomson et al., 1994; Treseder, 2013; Cheng et al., 2020), including under harsh conditions (e.g., Hachani et al., 2020; Klinsukon et al., 2021) or when competing with other plant species (e.g., Lin et al., 2015; Zhou et al., 2018). Our results suggest that under the pressure of interspecific competition, EcM and AM may improve the performance of *B. pendula* and *H. caespitosum* offspring in ecosystems where both species are present. In this context, the presence of a larger conspecific neighbor or, at least, of a plant with the same mycorrhizal type functioning as inoculum source seems to be essential for optimal seedling development during ecological succession of mixed plant communities on spoil banks, as has been described for other plant species and ecosystems (Nara, 2006a; Casanova-Katny et al., 2011; Zhenxing et al., 2019).

## Conclusion

Our study clearly demonstrates an asymmetric effect of the interaction of a pioneer herbaceous AM host and a woody EcM species on their mycorrhizal symbionts and colonization rates of seedlings. While *H. caespitosum* and associated AMF reduced abundance of soil EcMF and decreased mycorrhizal colonization of *B. pendula* seedlings, the EcM host did not have such effects on the AM plants and associated AMF. In natural conditions, this may favor *H. caespitosum* in competition with *B. pendula*, thus slowing the transition of vegetation from AM-dominated to EcM-dominated in early stages of succession on the studied spoil banks. The outcome of our study strongly supports the concept that EcM and AM should not be viewed simply as alternative plant adaptations that minimize niche overlap and foster coexistence of their hosts (Janos et al., 2013). Depending upon the taxa studied, they can positively or negatively influence intra- and/or interspecific plant competition (Moora and Zobel, 1996; Janos et al., 2013), thus codetermining plant community shifts in succession.

## Data Availability Statement

The original contributions presented in the study are included in the article/Supplementary Material, further inquiries can be directed to the corresponding author.

## Author Contributions

JR, PK, and JF designed the study. TK and NF performed material preparation, data collection, and analysis. TC performed the fatty acid analyses. NF wrote the first draft of the manuscript. MJ, PK, and JR contributed to previous versions of the manuscript. All authors read and approved the final manuscript.

## Funding

Financial support was provided by the Czech Science Foundation (grant GA19-04902S) and the long-term research development project RVO 67985939.

## Conflict of Interest

The authors declare that the research was conducted in the absence of any commercial or financial relationships that could be construed as a potential conflict of interest.

## Publisher’s Note

All claims expressed in this article are solely those of the authors and do not necessarily represent those of their affiliated organizations, or those of the publisher, the editors and the reviewers. Any product that may be evaluated in this article, or claim that may be made by its manufacturer, is not guaranteed or endorsed by the publisher.

## Footnotes

^1^ https://www2.dijon.inrae.fr/mychintec/Mycocalc-prg/download.html
